# Understanding the experiences of family, friends and carers attending Recovery Colleges: focus group study

**DOI:** 10.1192/bjo.2024.852

**Published:** 2025-03-11

**Authors:** Bryher Bowness, Sarah Bicknell, Lana Samuels, Yasma Osman, Vanessa Kellermann, Claire Henderson, Vanessa Lawrence

**Affiliations:** Department of Health Service & Population Research, Institute of Psychiatry Psychology & Neuroscience, King’s College, London, UK; South London and Maudsley NHS Foundation Trust, London, UK; Independent Researcher, UK; Birmingham & Solihull Mental Health NHS Foundation Trust, Birmingham, UK; Warwick Applied Health, University of Warwick, Coventry, UK; Independent Researcher, UAE

**Keywords:** Coproduction, family carers, mental health recovery, qualitative research, Recovery Colleges

## Abstract

**Background:**

Family and friends (family carers) provide substantial support to those with mental ill health, often affecting their own well-being. Subsequently, family carers have their own recovery journeys. Research highlights numerous benefits of attending Recovery Colleges, but whether these apply for family carers remains unexplored.

**Aims:**

We aimed to explore family carers’ experiences of attending Recovery Colleges across England, to understand current provision and how this might better include and support family carers.

**Method:**

Together with lived experience researchers, this qualitative focus group study used collaborative thematic analysis of online focus groups and interviews with family carers and Recovery College staff from across England.

**Results:**

We generated six superordinate themes: ‘The “carer” identity is not clearcut’, ‘Recovery ethos applies to family carers too’, ‘Power of lived experience’, ‘Educational focus is appealing’, ‘Family carers deserve recognition and provision’ and ‘Reaching out and fitting around family carers’. Attending Recovery Colleges developed family carers understandings and gave them skills to navigate services and support themselves and others, which furthered their own recovery journeys. Shared learning spaces were helpful, but participants felt these were not always oriented to include family carers. Our findings revealed ways Recovery Colleges could increase their relevance and accessibility to family carers.

**Conclusions:**

The unique characteristics of Recovery Colleges suited the recovery needs of family carers. However, more resources are needed to develop this potential and reach more family carers. Family carer co-researchers enriched our findings, and discussions with the Recovery College community furthered our recommendations for practice.

The term ‘carer’ is frequently used to describe a diverse group who provide substantial informal support to family or friends with disabilities or ill health. However, most do not identify as carers,^1^ referring to themselves instead as parents, partners, children, siblings or friends, and this term oversimplifies a dynamic and often reciprocal relationship.^2^ Here, we use the term ‘family carer’, where ‘family’ may be chosen, and includes friends, kin and neighbours. There are roughly 8.8 million family carers in the UK, and around 13% of these are caring for someone with a mental health difficulty.^3^ Since deinstitutionalisation and reduced formal mental health provision,^4^ family carers are increasingly relied on. They often report their own distress,^5^ and are at increased risk of social isolation and financial difficulty.^6^ Despite UK policy commitments,^7^ family carers continue to report feeling excluded and unheard by services.^8^ A consistent and holistic approach to their support is needed.^9^

Personal recovery in mental health is described as a journey of finding meaning in what has happened, becoming an expert in self-care, building a new identity and discovering resources to pursue goals.^10^ Influenced by the recovery education movement in the 1990s, Recovery Colleges aim to promote personal recovery while transforming wider mental health practice. Initially established in London in 2009, there are now roughly 221 Recovery Colleges worldwide,^11^ with 88 in England used by over 360 000 ‘students’.^12^ Mental health service users, family carers, staff and community members learn together in self-selected, free courses that are co-designed and co-delivered between experts-by-experience and experts-by-training. Colleges offer a variety of courses: one-off sessions, over numerous weeks, online and in person. Curriculum areas include understanding conditions, developing skills for recovery, and mind and body.

Benefits include increased self-esteem and social inclusion,^13^ and mental health staff report reduced anxiety and increased recovery-oriented practice.^14^ The 6–11% of students who identify as family carers (estimates from multiple Recovery College service evaluations; this figure is likely to be higher, given many students hold multiple roles or might not recognise themselves as carers) may experience Recovery Colleges differently, as they sometimes have contrasting understandings of mental health treatment and recovery.^15^ Close others are described as essential in many people’s recovery narratives,^16^ but they also have their own, albeit interrelated, recovery journeys.^17^ For both these reasons, recovery education may be beneficial for family carers. One Australian service evaluation found family carers voiced benefits to their well-being, the relationship with the person they supported and reduced internalised stigma.^18^ But to date, there has been no specific research into family carers’ experiences of Recovery Colleges.

## Aims

We aimed to explore family carers’ experiences of attending Recovery Colleges across England, to understand current provision and ways this might better support family carers.

## Method

### National survey

Four questions about provision for family carers were added to an online survey completed by 62 Recovery College managers in England in 2021 (conducted for RECOLLECT 2, a larger research programme investigating how Recovery Colleges can provide the most benefit to patients).^12^

### Focus groups/interviews

#### Towards coproduction

**Fig. 1:**
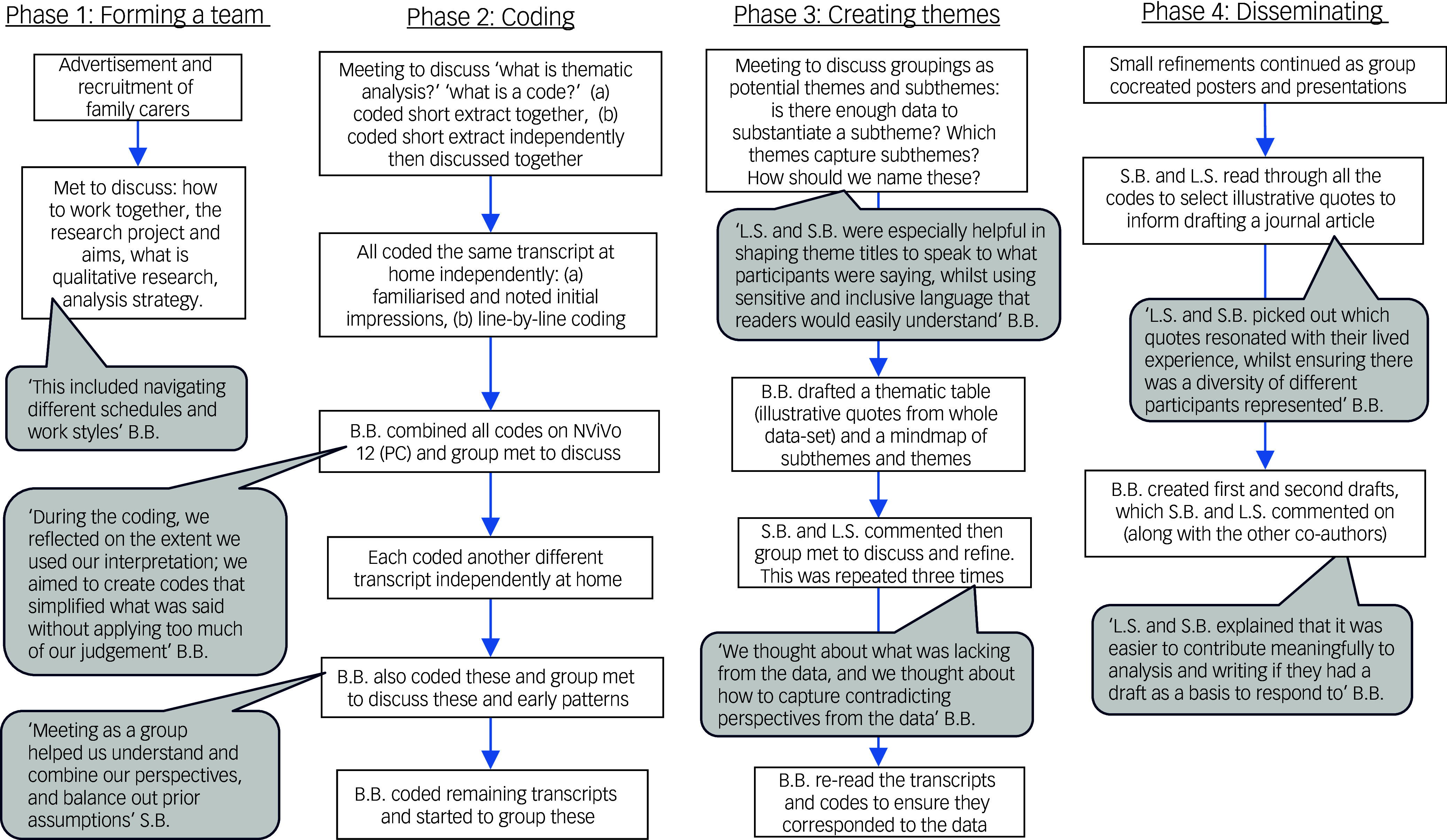
Flowchart of the collaborative data analysis process.

Aspiring to Recovery College’s ethos of coproduction, family carers were involved wherever possible in the decision-making and research (see Guidance for Reporting Involvement of Patients and the Public 2^19^ in the Supplementary Material). Combining diverse perspectives can have epistemological advantages for research,^20^ and involving affected communities in knowledge production be empowering.^21^ First, B.B. held four online meetings with a family carer researcher (Y.O.) to co-design the study methods. Then, two family carer researchers (L.S. and S.B.) joined B.B. to analysis and disseminate the findings (Fig. 1). Unfortunately, organisational considerations necessitated pragmatic adaptations to coproduction, which we navigated together.

#### Ethics

The authors assert that all procedures contributing to this work comply with the ethical standards of the relevant national and institutional committees on human experimentation and with the Helsinki Declaration of 1975, as revised in 2013. All procedures involving human participants were approved by North of Scotland Research Ethics Committee (REC 22/NS/0116, IRAS ID 3111832). All participants were given information sheets and offered a telephone call with the researcher before completing online consent forms.

During co-design, our family carer researcher drew on her lived experience to make participant materials easier to understand, suggest ways to sensitively signpost to further support, design reimbursement methods, and ensure we did not overpromise participants immediate service impact.

#### Design

Qualitative online focus groups were selected as a generative method where participants can explore as a group new ideas and perspectives. To maximise inclusivity, we offered individual interviews as alternatives (aiding scheduling difficulties, common with family carers^22^), and provided guidance/practice for those unfamiliar with online meeting software.

#### Sampling and recruitment

Informed by the RECOLLECT2 National Survey data,^12^ we approached managers from Recovery Colleges that varied according to characteristics we believed might influence family carer student experiences: years since opening, size, rurality and nominated family carer staff member (‘carer lead’). The family carer researcher wished to increase the likelihood of recruiting family carers from cultural groups often underrepresented in research, therefore we prioritised approaching Recovery Colleges with diverse catchment areas. Twenty-nine Recovery College managers agreed to publicise through emails, posters and word of mouth. Interested participants then emailed the researcher and completed a self-reported online eligibility questionnaire. Participants were eligible if they self-defined as family carers who had attended Recovery College (≥18 years), or if they worked for a Recovery College in a role/with an interest in family carers (‘carer leads’). Recruitment continued until data saturation (researchers noticed repetition and felt questions had been explored to substantial breadth and depth to meet the study aims).

#### Procedure

Written informed consent was obtained from all participants, who then completed a short demographics questionnaire and online poll with their availability. Participants were offered separate focus groups if they worked for a Recovery College and were not asked which Recovery College they came from, to encourage comfort in discussing challenges. Individual interviews were conducted by B.B., and focus groups were cofacilitated with another researcher with lived experience of mental health difficulties (V.K.). We used co-designed topic guides (Supplementary Materials) loosely to allow participants to initiate/explore ideas. Discussions were recorded, then transcribed by B.B. Facilitators held a reflective debrief after each group.

#### Data analysis

Our ontological stance was based in critical realism,^23^ where reality exists but is inevitably known through individual social lenses within specific contexts. This perspective allowed an experiential approach to the analysis, preferencing the lived experiences of participants while mapping similarities and suggesting shared social understandings.

We applied the steps of reflexive thematic analysis:^24^ familiarise, generate initial codes, search for themes, review themes, define themes and produce report. This is an accessible method to create succinct themes across multiple data-sets, and is well-suited to participatory approaches.^25^ Taking an inductive approach, we began by forming descriptive codes that captured our participants’ meaning, then brought our interpretation to the process of ordering, connecting and contextualising these into broader themes. These were reshaped, then defined through a process of iterative and collaborative cycles of discussion between the researchers: ‘synthesis was enabled by careful listening, lengthy and full discussion, a joint reflection on shared codes and ideas and an interweaving of our interpretations’.^26^

Collaborative data analysis is underpinned by the epistemological stance of perspectivism, with the assumption that bringing together multiple interpretations externalises assumptions and enables critical reflection. Analysis was conducted by three core co-analysts, a PhD student/mental health nurse (B.B.) and two family carer co-researchers (S.B. and L.S.). Combined, we also had experiences as mental health patients. Through regular discussions, we gradually got to know each other and reflected on how our positionality might have influenced the analysis. For example, previous experiences of attending and facilitating courses at Recovery College may have led to salience of more positive comments. Although resources limited full power-sharing, we aspired to make decisions as a group and create a process of learning from each other where all of our voices could shape the analysis (Fig. 1).

#### Interactive webinar

We presented our findings in an online webinar to approximately 80 people from a variety of Recovery Colleges worldwide, health and research organisations, and family carers, who we had recruited through online communities of practice and research networks. Based on themes from our focus groups/interviews, we used an interactive online whiteboard to gather audience responses to four questions relating to how our findings could be implemented by Recovery Colleges to support family carers.

## Results

### National survey

Summarised results regarding Recovery College provision for family carers are detailed in Table 1. Although the majority of Recovery Colleges had offered courses specifically around caring, only a third had a designated carer lead.

**Table 1:** Recovery College provision for family carers in England in 2021

Questions in the RECOLLECT 2 National Survey^15^	Total ‘yes’
Who is your Recovery College for? • Informal carers (e.g. family, friends) of people with mental health issues?	58/62 (93.5%)
If yes (*n* = 58):	
• Do you routinely monitor whether students are carers?	42/58 (72.4%)^a^
• Do you have a designated carers’ lead (someone who has a dedicated role to support informal family/friend carers) at your Recovery College?	18/58 (31.0%)^a^
• In the past 2 years have you run a course specifically for carers or caring for someone with mental health issues?	42/58 (72.4%)^a^

a. Percentage calculated of those Recovery Colleges that are open to carers (*n* = 58).

### Participants

Between December 2022 and February 2023, we conducted five focus groups and five interviews, with a total of ten participants who were carer leads/staff at Recovery Colleges, and 13 family carers who had attended as students (*n* = 23). Of the 16 participants who completed the demographic questionnaire, the majority were White British and female, with varying relationships to those with a range of diagnoses (Table 2).

**Table 2:** Participant demographics

Demographics questionnaire (participants answered in their own language)	Total (*n* = 16)
Gender	*n* = 16
Female	9
Male	5
Non-binary	2
Ethnicity	*n* = 16
White British	10
White European	2
Latin American	1
Indian	1
African	1
White Mixed	1
What is your relationship with the person you support/supported? If multiple people, please list all that apply	*n* = 23
Parent	8
Spouse/partner	6
Friend	3
Child	2
Sibling	2
Other family member	2
What is the mental health problem that the person you support/supported experiences/experienced? If multiple, please list all that apply	*n* = 37
Depression	7
Anxiety (including social anxiety)	7
Personality disorder (including borderline/emotionally unstable)	6
Psychosis	4
Bipolar disorder	2
Eating disorder (including anorexia)	2
Attention-deficit hyperactivity disorder	2
Self-harm/suicide	1
Autism	1
Intellectual difficulties/neurological problems	1
Dementia	1

### Themes

We developed six overarching themes (Table 3): The ‘carer’ identity is not clearcut, Recovery ethos applies to family carers too, Power of lived experience, Educational focus is appealing, Family carers deserve recognition and provision, and Reaching out and fitting around family carers. These relate to the elements of Recovery College that family carers found helpful, the benefits they experienced from attending and the ways provision could be improved.

**Table 3:** Overview of themes from focus groups/interviews with family carers and staff/carer leads^a^

Theme	Subthemes
The ‘carer’ identity is not clearcut	• People don’t always see themselves as ‘carers’ • Students can be many things at once – family, professionals and people with mental health difficulties
Recovery ethos applies to family carers too	• Family carers highlighted the trauma of caring • Recovery Colleges helped family carers realise recovery also applies to them • Recovery colleges helped maintain, reshape or ‘save the relationship’ • Recovery Colleges further recovery
Power of lived experience	• Co-production deepened understanding • Family carers gained from learning and sharing with other students
Educational focus is appealing	• Family carers want to develop their understanding • Recovery Colleges were supportive while maintaining an educational focus
Family carers deserve recognition and provision	• Family carers are rarely made the priority • Courses focused on family carers are ‘brilliant’ • Recovery Colleges should consider family carers in all courses
Reaching out and fitting around family carers	• Recovery Colleges need more ways to raise awareness • Recovery Colleges should fit around family carers’ lives

a. Not described in order of importance, but rather in a narrative structure.

#### The ‘carer’ identity is not clearcut

People don’t always see themselves as ‘carers’. The label was associated with physical disability and personal care, whereas supporting someone with their mental health involved less tangible tasks, like being on-call in a crisis or taking on familial responsibilities. People’s mental health needs were often fluctuating, ‘*I didn’t do that much caring really, I mean, it depends with him’* (C, family carer, interview3); some participants felt ‘carer’ implied passivity or disability of the person they supported so was not a very recovery-oriented word. There was debate as to whether *‘using the word carer is a barrier’* (A, carer lead, fg2) for people coming to Recovery Colleges, and more inclusive language, such as ‘supporter’, might be preferable. Alternatively, other staff felt it was important to help students recognise they were carers as this can enable service access, and so offered courses on ‘Who is a Carer?’

Students can be many things at once – family, professionals and people with mental health difficulties. Consequently, monitoring the number of carers was challenging, and some staff felt this was at odds with the Recovery College principles of leaving labels at the door: *‘a student is a student is a student’* (K, carer lead, fg2). Participants described how roles within their relationships were dynamic and interactional; supporting each other in *‘the mechanism of joint caring’* (M, carer lead, fg1). Some people had previous mental health experience which gave them skills to support family/friends, whereas others had become unwell themselves following periods of caring. Some participants approached Recovery Colleges initially for other reasons before realising it also helped them in a caring role. Within the Recovery College space, participants found ways to navigate these multiple identities, *‘I put different hats on, take them off, but I’m still me, I’m not a different person’* (T, carer lead, fg1).

#### Recovery ethos applies to family carers too

Participants highlighted the trauma of caring, which they carried with them in Recovery College spaces: *‘when our loved ones have trauma, so do we. And I don’t mean that in a selfish way. I just mean in a holistic way for everybody’s recovery’* (S, carer lead, interview 4). We heard distressing experiences, such as supporting family members through suicide attempts or psychosis, and a range of emotions such as guilt, helplessness, frustration and feeling ‘utterly terrified’ (we have tried to use the words of the participants to describe our themes wherever possible, in addition to providing illustrative quotes. We have attributed them to their full original quotes in the Supplementary Material). For some, their role became ‘*all-consuming 24/7, all you do is think about it’* (N, family carer, fg5), which could be exhausting, ‘suffocating’ and lonely. Some family carers had found Recovery College at a stage of desperation or crisis, whereas others were in the aftermath beginning to process their experiences. The ‘right time’ for Recovery College *‘everyone is different. So every single person maybe needs support at a different stage’* (L, family carer, interview 5).

Recovery College helped family carers realise recovery also applied to them: ‘*It’s been a journey for me…going on Recovery College courses… over time made me realise that I do need to open up a bit more. There is stuff that I do need. I have to go on my own recovery journey’* (N, family carer, fg5). Participants described learning about themselves as well as the person they supported, and having ‘light-switch moments’ when they realised *‘Gosh, this is relevant to you’* (C, family carer, interview 3). Thus, attending Recovery College had been a ‘turning point’ in their caring journeys.

Recovery College helped maintain, reshape or ‘*save the relationship’* (K, family carer, fg3). Recovery College staff tried to ‘shift that balance a little’ to help family carers think about themselves: *‘it was the first time I’d really thought, well yea, you know, perhaps I should build some of my life around me rather than building it around my son’* (C, family carer, interview 3). Participants began to recognise their limits and realised trying to fix everything or be a ‘perfect’ carer was not sustainable. Staff and students frequently used metaphors such as putting on their oxygen mask before looking after others: *‘if we don’t recover, we can’t support’* (S, carer lead, interview 4). Courses like ‘Caring for Carers’ emphasised that family carers also need and are worthy of help, and provided self-care strategies such as gratitude journals. Participants had attended other courses such as ‘Creative Doodling’ or ‘Comedy School’, taking time out was important; *‘not to be serious, you know, with us with carers’* (S, carer lead, interview 4). Recovery College *‘fires up your batteries. So you’re, you know, able more able to care for the person you are caring for’* (K, family carer, fg3).

Recovery Colleges furthered recovery in a holistic sense for both family carers and the people they supported: *‘you never know with recovery how it just ripples out’* (K, carer lead, fg2). By learning to manage their own mental health, participants developed an authentic understanding of recovery, enabling them to then journey alongside the person they supported. Participants expressed needing to move forward, try something new and *‘get on with my life’* (A, family carer, fg4), rebuilding an identity beyond their caring role: *‘separation is important – who am I?’* (T, carer lead, fg1). Courses, such as ‘Couch to 5k’, gave participants a sense of achievement and learning skills boosted confidence and empowered them to *‘actually make some massive changes’* (M, family carer, fg5). They took up new opportunities like becoming autism awareness champions or take peer worker courses. Staff framed this as helping family carers in ‘taking back control’, which may have been lost when services intervened without including them in the care of their family member/friend. Being able to *‘turn this around a bit. It’s kind of given me hope’* (D, family carer, fg5) and see a *‘light at the end of the tunnel,’* (K, family carer, fg4).

#### Power of lived experience

Lived experience of both the tutors and the other students was ‘crucial’ – *‘It is education. But it’s not. It’s much more human’* (K, carer lead, interview 6) – and comes from a unique angle to the information provided by clinical services.

Co-production and co-delivery between clinicians and people with lived experience allowed students to ‘*ask both how it is from the person that you know is a professional, and someone involved’* (L, family carer, interview 5). Tutors with lived experience of caring ‘resonated’ with participants, who felt ‘deeply understood’. Hearing from people further along in their recovery provided an opportunity to ask questions they may not be able to voice with their relative or friend. This *‘put my mind at ease’* (K, family carer, fg3), creating ‘*a sense that things can get better’* (S, carer lead, interview 4).

Family carers gained so much from learning and sharing with other students. One participant asked for more opportunities for students to *‘share some of their life experiences and like what they’ve done to get to where they are’* (C, family carer, fg5). The shared learning environment exposed students to different perspectives, but also helped them feel *‘they weren’t the only one’.* Recovery Colleges created a safe space away from stigma or judgement, which empowered them to *‘feel confident to talk about…your loved one’s mental health’* (R, family carer, fg4). In-person courses, and those running over multiple weeks, allowed students to get to know each other and provided a ‘mutually supportive’ community. Students acknowledged each other’s progress providing a further source of hope.

#### Educational focus is appealing

Family carers wanted to develop their understanding of what was happening, what the future might hold, and *‘techniques and tools to help manage what might come up’* (K, family carer, fg4). Pressure on formal services, exacerbated by factors such as the COVID-19 pandemic, had led to *‘a gap that could be well supported by Recovery Colleges’* (R, carer lead, fg2), and participants reported rarely receiving information or support when their relative/friend became unwell. Knowledge of mental health and recovery also deepened their empathy for the person they were supporting, helping the relationship. Attending Recovery College appealed as a proactive step: ‘*I’m going to actually do something. I’m not just gonna go and sit and drink coffee’* (M, family carer, fg5). Carer leads informed carers about their rights and how the system worked to give them *‘more confidence when they approach services’* (A, carer lead, fg2), ‘*I cannot say I know everything about mental health right now… [after] only week five or six courses, but I feel less intimidated*’ (R, family carer, fg4).

Recovery Colleges were supportive while maintaining an educational focus, a challenging balance. Family carers did not have to disclose personal experiences or worry about their relative/friend being identified, which appealed to those who *‘may find it hard to be… they would be scared of being vulnerable’* (S, carer lead, interview 4). The comfort of the educational structure *‘opened up lots of the discussions about how people actually are’* (S, family carer, fg3). Although those who shared found the encouraging response of the group therapeutic, others recognised that oversharing by other students ‘*was not helping, or it was maybe even harming me’* (C, family carer, interview 3), leading them to compare themselves to others or feel pressure to be a better carer. Emotions of family carers who were dissatisfied with their relative’s/friend’s care or who lacked therapeutic outlets elsewhere were described as barriers to learning. To maintain a safe learning environment, Colleges stopped running family carer forums and coffee mornings, which had left more *‘space to express that frustration and possibly that sadness that comes with it all’* (K, carer lead, interview 6). While trying to remain *‘educators that care’* (K, carer lead, interview 6), tutors tried to *‘steer [discussions] to giving each other information and think of ideas’* (J, carer lead, fg1). They offered individual signposting, and one College partnered with a therapy and respite carers’ charity, enabling courses to become spaces to reflect and an *‘opportunity of kind of turning negatives into positives’* (S, carer lead, interview 4).

#### Family carers deserve recognition and provision

Family carers were rarely made the priority within Recovery Colleges: *‘kind of the last on the list’* (D, family carer, fg5). Despite publicising their courses as mixed, our participants reported that some staff were *‘surprised that a carer was in the room’* (N, family carer, fg5). ‘*Sometimes there is no mention of carers… the assumption is that the person who is recovering is alone’* (R, family carer, fg4). This led some family carers to wonder whether they were supposed to be there, and to withhold their contributions: *‘just there to observe and to learn’* (K, family carer, fg3). Staff were concerned these carers might not *‘make use of the space because again they’re focusing on the people who supposedly need it more than them’* (A, carer lead, fg2).

Courses focused on caring are ‘brilliant’, but Recovery Colleges should consider family carers in all courses. To help welcome family carers, some Recovery Colleges had carer-only courses, and some had developed curriculums around family carer interests, coproduced with external specialist services (like psychosis or older persons teams) or local carer support groups. But foremostly, participants found learning together with staff and patients to be beneficial, and enjoyed attending a range of courses. Thus, Colleges should *‘always be thinking, well, what would a carer make of this? What would a carer want to know?’* (S, carer lead, interview 4).

Carer leads described their longstanding efforts to drive change: *‘you don’t know how many years I’ve been banging on about “Remember the families!”’ (J, carer lead, fg1).* They highlighted the disparity between Recovery Colleges and the need to learn from each other. However, *‘you also have to be realistic in what you can offer, because the worst thing for me as a carer, if somebody promised me something and couldn’t do it – we’ve been let down too many times’* (S, carer lead, interview 4). For all Colleges, funding was an ongoing barrier, so improvement *‘trickles down to people and to families is, let’s say, very slow’* (A, carer lead, fg2).

#### Reaching out and fitting around family carers

Participants *‘would just like the opportunity to be given to all carers’* (D, family carer, fg5), but felt not enough were attending.

Recovery Colleges need more ways to raise awareness because participants reported ‘*you have to be a proactive carer that goes looking for it, rather than them come looking for you’* (N, family carer, fg5). Even most patients had never heard of Recovery Colleges. There was also concern that family carers from marginalised communities were less likely to attend: ‘*you’ve got that huge hidden demographic that we’re trying to access’* (D, carer lead, fg1). Participants made many suggestions for ways to improve general publicity (Table 4), but also called for explicit carer-specific advertisements. It was felt that other services and clinicians were *‘pivotal people worth targeting or linking with’* (K, carer lead, fg2), as they shared responsibility for signposting family carers to the Recovery College.

**Table 4:** Suggestions for how Recovery Colleges could implement findings, to improve relevance of their provision for and inclusion of family carers

Questions posed in webinar	Examples suggestions from focus group/interview participants	Example webinar audience suggestions (*n* indicates number of responses)
How can we make Recovery Colleges more relevant for family carers?	• Family carers on quality assurance panels • More trainers with caring experience • Family carer videos/quotes in courses • Highlight family carer courses on prospectus • Coproduce with specialist services/carer charities • Find out what local carers want through support groups • Carer champions within Recovery Colleges • Connect with National Health Service carer leads and Triangle of Care initiatives	• Reflect on feedback forms • Monitor number of family carers – this might encourage funding • Increased involvement in co-production • Design tailored family carer courses – have courses on understanding experience of family carers and how they can support themselves, e.g. compassion fatigue, trauma stewardship, burnout • Ask carer support groups what they might want – take time to listen to family carers • More art and well-being courses • Increased understanding of recovery journey for family carers, and courses highlighting how recovery principles link to family carers • Specific family member courses, e.g. adult sibling, father • Question and answer formats – conversations focused • Lots of information (particularly about practical support) available (*n* = 63)
How can we make it easier for family carers to attend Recovery Colleges?	• Courses out of working hours • Online, webinars, podcasts, handouts • Social workers, volunteers or peer support workers can go with family carers • Breaks in courses to do tasks • Coordinate with carers charities who can provide therapeutic support and respite activities	• Ask family carers who enrolled but did not attend what the barriers are and what might have helped? • Doodle polls of availability to schedule courses • Include whole families • Opportunity for taster sessions or virtual introduction to the trainers • Shorter courses are easier to attend • Changing funding structures to allow online courses to be available across the UK (not all family carers are local) • Include this question on evaluation forms • Offer emotional support as talking can trigger sadness • Funding to help family carers access online courses (*n =* 56)
How can Recovery Colleges reach diverse communities?	• More diversity among trainers • Language around caring and mental health, e.g. ‘well-being’ • Outreach into the communities • Links with community leaders • Providing IT skills classes and access to computers to address digital exclusion	• Translation in local languages • Publicise at faith groups, sports clubs • Publicise through interviews on cultural radios • Offer courses to young carers • Co-produce with community leaders • Language, e.g. ‘well-being’/‘hope’ carry less stigma • Analyse enrolment data to identify underrepresented groups • Arts courses carry less stigma • Physically present in their communities • Base courses in non-clinical settings (*n* = 51)
How can Recovery Colleges raise awareness?	• Include stories/quotes from family carers in Recovery College publicity material • Adverts on buses, shopping centres, chemists, gyms, libraries • Other services need to promote Recovery Colleges, e.g. general practices, carer support groups, those conducting Carer’s Assessments, helplines, hospitals, accident and emergency departments, social prescribers • Renaming the College to make it sound more relevant, e.g. ‘Hope College’ • Analyse and create a marketing/publicity strategy • Publicise on multiple social media platforms • Ensure it is clear the range of courses and breadth that Recovery Colleges offer	• UK wide promotional campaign for Recovery Colleges • Recovery Colleges need more funding • Marketing lead in Recovery Colleges • Language ‘supporters/families’ • Talks in workplaces • Promote through social prescribers • Publicise through education teams • Building connections with different services • Showcasing events • Open day or coffee morning to welcome family carers • Whole system needs more family-inclusive practice (*n* = 31)

Recovery Colleges should fit around family carers’ lives, as attending Recovery College is not always easy. Some family carers had been socially isolated, or were worried about groups, and they described ways this initial apprehension could be reduced (Table 4). With a lot going on in their lives, family carers were sometimes *‘so absorbed with that, that I sometimes don’t take on board the other things that have been said’* (C, family carer, interview 3), requiring simple content in digestible chunks. Busy family carers found one-off courses and sessions outside working hours more convenient. Online courses were easier to *‘incorporate into your routine’* (C, family carer, fg5); some even fitted other tasks into the breaks. Moreover, not all family carers could travel because of cost, disability and distance. Some Colleges offered their caring curriculum as recorded webinars or e-learning modules. However, these formats came with technical difficulties and the risks of digital exclusion. Plus, many family carers wanted the social connection of classroom-based courses. There was no ‘one size fits all’ that suited family carers: *‘you can kind of increase that inclusivity is just to offer the variety of give and then something for everyone’* (M, family carer, fg8), but this flexibility would require more funding.

#### Interactive webinar

Based on the above themes, the webinar audience provided 201 suggestions for how Recovery Colleges could help more family carers attend, increase relevance for them, raise awareness and include diverse communities. Their summarised responses, alongside suggestions from the focus groups/interviews and ideas from our family carer researchers, are detailed in Table 4.

## Discussion

Our findings provide an initial understanding of family carers’ experiences of attending Recovery Colleges, which we discuss in relation to their caring role and well-being journeys, and also their potential implications for practice.

The participants’ accounts reflected the relational model of family carer recovery journeys, which describes three parallel and interdependent dynamic processes.^17^ Recovery College courses increased their confidence in supporting the person they cared for, helped their caring relationship and supported the family carers’ well-being. Participants explored how these benefits interacted. Echoing existing research, accessing mental health knowledge, support from others with similar experiences, and focusing on self-care reduced distress^27^ and nurtured hope^28^ among the family carers in our study.

Family carers can often experience guilt engaging in self-care,^29^ but courses at the Recovery College helped them realise that managing their own needs was necessary to sustainably care for others. To avoid perpetuating a societal discourse that maintains the reliance on unpaid carers in the context of reduced formal service availability,^30^ Recovery College staff can support family carers to rediscover their self-worth and identity beyond their caring role (something our participants reported). Critics argue that providing information without availability of resources could inadvertently disempower family carers.^31^ However, learning together created a sense of connectedness, arguably lacking elsewhere in the increasingly individualised care system.^30^ Moreover, Recovery College staff aimed to inform family carers of their rights, increase their confidence to engage with services and signpost them to therapeutic support, complementing rather than substituting formal provision.^18^

Family carers in this study, like many others,^32^ had struggled to find help elsewhere. Although there is a substantial evidence base for family psychoeducation groups,^33^ these are ‘startlingly unimplemented’.^34^ Our participants reported similar perceived benefits to family psychoeducation, such as increased confidence, reduced stigma and improved well-being,^35^ but Recovery Colleges had fewer barriers to access.^36^ Rather than a standardised offer, students self-select courses to suit their stage in the caring journey. There is no referral needed, and family carers can access the Recovery College independently from the person they support.

Although generally, students find the co-learning space at Recovery Colleges beneficial,^37^ the potential for tension arising from differing views between family carers and patients should be considered when increasing family carer involvement in recovery spaces. Some participants believed they should defer to patient voices in the classroom, or felt more comfortable in family carer-only courses. However, after attending courses specifically for family carers, most participants went on to attend the whole range of courses, where they found a safe space to learn from students with similar conditions to those they supported. Adopting the shared primary identity of ‘student’ enabled the exchange of diverse perspectives to result in growth. Recovery Colleges have the potential to increase empathy and reciprocal understanding, to reduce stigma and family conflict^38^ and promote ‘mutual recovery’.^39^

### Strengths and limitations

The family carer researchers brought invaluable lived experience to the design and analysis of this study, attended to diversity in the data and refined theme labels and definitions. Ideally, they would have cofacilitated the focus groups, but bureaucratic requirements unfortunately prevented this. All three analysts compared the data to their own experiences of working in and/or using Recovery Colleges, increasing real-life applicability of the findings.

This was the first multi-site study to explore family carer perspectives, but we did not ask participants to identify which Recovery College they had attended, and so cannot state how representative this sample was or explore relationships with organisational differences. The interactive webinar provided further opportunity to check coherence with a wider audience, and built momentum and forged connections between carer leads nationally.

Despite recruiting from diverse catchments, participants from our sample who completed the demographic questionnaire were majority female and White British (Table 2). This is typical of patient students at Recovery Colleges,^40^ and reflects bias in participation in web-based surveys.^41^ Using multiple and more inclusive outreach strategies may have reduced this. Participants argued for earlier outreach for family carers; following our dissemination we learnt from the Wakefield Recovery College manager how young carers had championed their Discovery College. Although we only included adult Recovery Colleges, this indicates a future research avenue.

Our purposefully broad definition of ‘family carer’ meant many of our participants held multiple identities, and subsequently, several of our findings likely resonate with experiences of Recovery College students generally. Although we specifically gave space to the family carer perspective, Recovery College students more widely should ideally be consulted before recommendations to increase involvement of family carers are implemented.

### Implications for practice

A previous study found that of 22 Recovery Colleges surveyed, only two identified increasing their provision for family carers as a future priority.^42^ Through initiatives similar to the numerous suggestions outlined in our study (Table 4), ImROC (Implementing Recovery-Oriented Care; https://imroc.org/) urged Recovery Colleges to further involve family carers^43^ so as to remain faithful to their defining dimension of inclusivity.^10^ Increasing involvement of family carers may have additional transformational effects (for example, helping healthcare staff’s ‘carer awareness’). Thus, the Recovery College model can be seen to support the partnerships between service users, family carers and clinicians central to the Triangle of Care policy initiative.^44^ Quantifying benefits of Recovery Colleges for family carers, and the effects of this for patients and mental health staff too, would strengthen arguments for much needed funding.

## Supporting information

Bowness et al. supplementary material 1 Bowness et al. supplementary material

Bowness et al. supplementary material 2 Bowness et al. supplementary material

Bowness et al. supplementary material 3 Bowness et al. supplementary material

Bowness et al. supplementary material 4 Bowness et al. supplementary material

## Data Availability

The data that support the findings of this study are available from the corresponding author, B.B., upon reasonable request.

## Supplementary material

The supplementary material is available online at https://doi.org/10.1192/bjo.2024.852
