# Supplementary material for: Understanding the experiences of family, friends and carers attending Recovery Colleges: focus group study
Source: BJPsych Open. 2025 Mar 11;11(2):e43. doi: 10.1192/bjo.2024.852 (PMC12001944; doi:10.1192/bjo.2024.852)
Supplement: Bowness et al. supplementary material 1 — Bowness et al. supplementary material [file S2056472424008524sup001.docx]

**Supplementary Material 1**

GRIPP2 short form

| **Section and topic** | **Item** | **Reported page** |
| --- | --- | --- |
| 1: Aim | Report the aim of PPI* in the study | Para 2, p4  Para 5, p5 |
| 2: Methods | Provide a clear description of the methods used for PPI in the study | Para 2, p4,  Figure 1, p5 |
| 3: Study results | Outcomes—Report the results of PPI in the study, including both positive and negative outcomes | Para 2, 3, p4  Para 5, p5  Para 2, p12  Figure 1, p6 |
| 4: Discussion and conclusions | Outcomes—Comment on the extent to which PPI influenced the study overall. Describe positive and negative effects | Para 2, 3, p4  Para 5, p5  Para 2, p12  Figure 1, p5 |
| 5: Reflections/critical perspective | Comment critically on the study, reflecting on the things that went well and those that did not, so others can learn from this experience | Para 2, p12  Figure 1, p5 |

**PPI* patient and public involvement

Staniszewska S, Brett J, Simera I, Seers K, Mockford C, Goodlad S, Altman DG, Moher D, Barber R, Denegri S, Entwistle A. GRIPP2 reporting checklists: tools to improve reporting of patient and public involvement in research. bmj. 2017 Aug 2;358.
