# Supplementary material for: Understanding the experiences of family, friends and carers attending Recovery Colleges: focus group study
Source: BJPsych Open. 2025 Mar 11;11(2):e43. doi: 10.1192/bjo.2024.852 (PMC12001944; doi:10.1192/bjo.2024.852)
Supplement: Bowness et al. supplementary material 2 — Bowness et al. supplementary material [file S2056472424008524sup002.docx]

**Topic guide for carer leads of a Recovery College**

Go round and briefly introduce yourself, and what your role is within the Recovery College. If you are happy to, please share a little bit about this role (how it was started, what it entails …)

How did you become involved in the Recovery College?

- What was your motivation for involvement with the Recovery College?
- What has your experience of the Recovery College been so far?

What courses do family/ informal carers^[[1]](#footnote-1)^ at the College generally attend and why?

Are there provisions and facilities specifically for family/ informal carers at your College?

- What do you think about courses specifically for family/ informal carers?

What do you think family/ informal carers find helpful about the Recovery College?

What are the needs of family/ informal carers who attend the Recovery College?

- Are there any challenges as a family/ informal carer attending the Recovery College?

How do you think the experience of the Recovery College as a family/ informal carer differs from service users or staff who use the College?

If you are a family carer yourself, how do you bring your carer perspective to your role in the Recovery College?

- Are there challenges that being a carer brings to your roles in the Recovery College?
- Has how you feel about being a carer/ supporter of someone with mental ill-health changed since your involvement with Recovery College?

What does Recovery mean to you? Has this changed since your involvement with the Recovery College? *please write this on the online Google Jamboard (the link to which is in the chat).^[[2]](#footnote-2)^*

How does the Recovery College differ to other support/ resources for family/ informal carers?

How could the Recovery College support family/ informal carers better?

- Are family/ informal carers listened to in the Recovery College?
- What might attract more carers?
- Are there other things that you would like to see courses on?
- Are there other opportunities Recovery Colleges could offer?
- What could Recovery Colleges do differently?

1. Terms such as ‘carer’, ‘family caregiver’, ‘supporter’ were used interchangeably to suit what the participants used themselves and what they felt comfortable with [↑](#footnote-ref-1)
2. Removed in future iterations due to time constraints and relevance to the topic [↑](#footnote-ref-2)
