# Supplementary material for: Understanding the experiences of family, friends and carers attending Recovery Colleges: focus group study
Source: BJPsych Open. 2025 Mar 11;11(2):e43. doi: 10.1192/bjo.2024.852 (PMC12001944; doi:10.1192/bjo.2024.852)
Supplement: Bowness et al. supplementary material 3 — Bowness et al. supplementary material [file S2056472424008524sup003.docx]

**Topic guide for family carers**

How did you first become involved in the Recovery College?

- *How did you hear about it?*
- *What were your motivations for attending? What did you hope to get out the Recovery College?*
- *Was it to accompany the person you support to a course? Or to attend a course for your own mental health? Or was it to attend a course to assist you in your caring role?*

What are your views on the different types of courses offered at the Recovery College?

- What courses are specifically helpful for family/ informal carers^[[1]](#footnote-1)^?
- Are there courses that are not so helpful for family/ informal carers?

Can family/ informal carers get involved in the Recovery College in other ways?

How do you think the Recovery College can benefit people with mental health challenges?

How do you think the Recovery College can benefit family/ informal carers?

Have you found any benefit from attending the Recovery College? If so, would you be willing to share these?

- What has contributed to these benefits?
- Have these benefits subsequently impacted the person you care for?

What are the potential challenges of attending the Recovery College?

- Is it easy to access?
- What support does the Recovery College provide any support to overcome these challenges?

Are there any ways that family/ informal carers might have negative experiences of attending Recovery Colleges?

How do you think your experience of the Recovery College as a family/ informal carer differs from service users or staff who use the College?

How does the Recovery College differ from other support that is available for family/ informal carers?

Are there ways you feel the Recovery College could support family/ informal carers better?

- Are family/ informal carers listened to in the Recovery College?
- What might attract more family/ informal carers?
- Are there other things that you would like to see courses on?
- Are there other opportunities Recovery Colleges could offer?
- What could Recovery Colleges do differently?

1. Terms such as ‘carer’, ‘family caregiver’, ‘supporter’ were used interchangeably to suit what the participants used themselves and what they felt comfortable with [↑](#footnote-ref-1)
