# Supplementary material for: Understanding the experiences of family, friends and carers attending Recovery Colleges: focus group study
Source: BJPsych Open. 2025 Mar 11;11(2):e43. doi: 10.1192/bjo.2024.852 (PMC12001944; doi:10.1192/bjo.2024.852)
Supplement: Bowness et al. supplementary material 4 — Bowness et al. supplementary material [file S2056472424008524sup004.docx]

**Supplementary Material 4**

Quotes influencing descriptions of our themes in full manuscript

| **Subtheme** | **Quote and participant** |
| --- | --- |
| **Participants highlight the trauma of caring** | “I was just *utterly terrified*” K (family carer, fg4)  “is like your life *suffocating* because I want to get on and do different things” A (family carer, fg4)  “it has to be at the *right time*, I think, and that’s not always easy to identify” S (carer lead, interview4) |
| **Recovery College helped family carers realise recovery also applied to then** | “real *light switch moment* in that it helped me recognise I was a perfectionist” C (family carer, interview3)  “because it can really offer you that *turning point* to do much more than just support you,” M (family carer, fg5) |
| **Recovery College helped maintain, reshape, or “save the relationship”** | “so we're always *trying to sort of shift that balance a little* bit and sort of thinking, OK, can we think about the relationship or can we think about you?” A (carer lead, fg2)  “that’s one of my things I’ve learned from the Recovery College about how much of a perfectionist I am…I think I tried to be perfect, and I could only keep that up for so long… Because I *have to be a perfect mother* as well, you see, but I’m not, but that’s what I was striving for, and of course I suppose perfect mothers don’t produce sons who try to take their life,” C (family carer, interview3)  *“Put your oxygen mask on first*, like if you're not OK and well in this situation, you're not gonna be able to help your husband, so that was all good stuff.” K (family carer, fg4) |
| **Recovery Colleges furthered recovery** | “That's what we do, offer hope, don't we? And *taking back control* and opportunity. And that’s something we stress in our carers course,” S (carer lead, interview4) |
| **Power of lived experience** | “I think the co- production with lived experience is *crucial*” D (family carer, fg5) |
| **Coproduction deepened understanding** | “that really *resonated* with me” K (family carer, fg4)  “she clearly had empathy, had lived experience, *understood deeply* what we were talking about,” N (family carer, fg5) |
| **Family carers gained so much from learning and sharing with other students** | “after sort of hearing other people’s stories, you realise that *you’re not the only one* in the situation,” K (family carer, fg3)  “we got to know each other and were *mutually supportive*” C (family carer, interview3) |
| **Courses on caring are ‘brilliant’, but Recovery Colleges should consider family carers in all courses** | “It was *brilliant* doing that, that that course for me,” K (family carer, fg4) |
| **Recovery Colleges need more ways to raise awareness** | “what I noticed about the opportunity for carers is that even the people that have mental health problems don’t know about the recovery college.” S (family carer, fg3) |
